# Supplementary material for: Secretion of Interleukin 6 in Human Skeletal Muscle Cultures Depends on Ca2+ Signalling
Source: Biology (Basel). 2023 Jul 7;12(7):968. doi: 10.3390/biology12070968 (PMC10376320; doi:10.3390/biology12070968)
Supplement: Supplementary file 1 [file biology-12-00968-s001.zip › biology-2149171-supplementary figure 1.pdf]

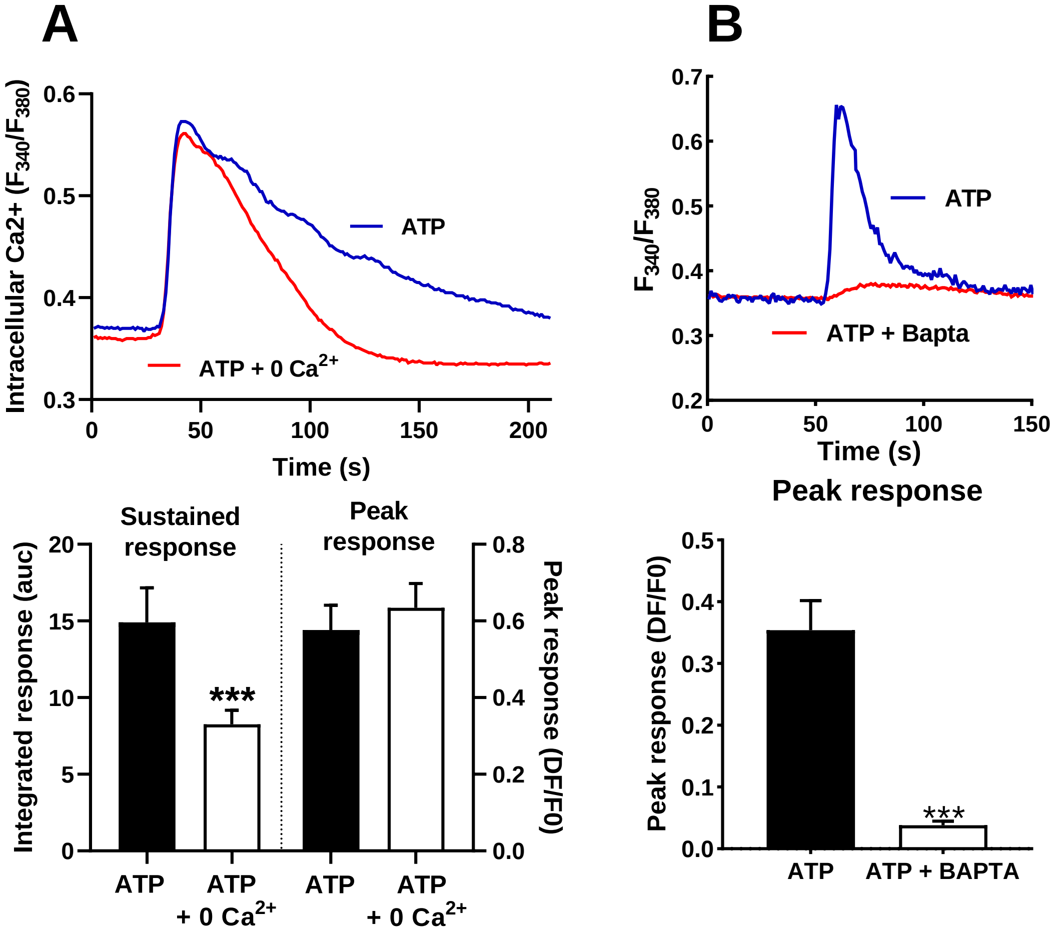

S1. Effect of removal of extracellular Ca2+ in the [Ca2+]i response to ATP.

A. 500 µM ATP was applied in normal medium (Tyrode solution containing 1.8 mM Ca^{2+}) or in a low Ca2+ medium (0Ca^{2+} added plus 1 mM EGTA) after 15 min pretreatment with the same medium. Representative average trace of 12 (ATP) and 16 (ATP+0Ca^{2+}) cells. Bottom panel compares the integrated response (area under the curve for 180 seconds, arbitrary units) and the peak response (DF/F0) of the cells represented in A. *** p<0.005, respect ATP, t test.

B. Representative trace of the ATP response in control cells and in cells loaded with Bapta-AM (20 min, 10 µM). Bottom histogram compares average peak response for treated and control cells. *** p<0.005, t test. n=25 (ATP) and 55 (ATP+Bapta) cells.
