# Supplementary material for: Secretion of Interleukin 6 in Human Skeletal Muscle Cultures Depends on Ca2+ Signalling
Source: Biology (Basel). 2023 Jul 7;12(7):968. doi: 10.3390/biology12070968 (PMC10376320; doi:10.3390/biology12070968)
Supplement: Supplementary file 1 [file biology-12-00968-s001.zip › biology-2347404-original blott figures/Suppl Fig 2.pdf]

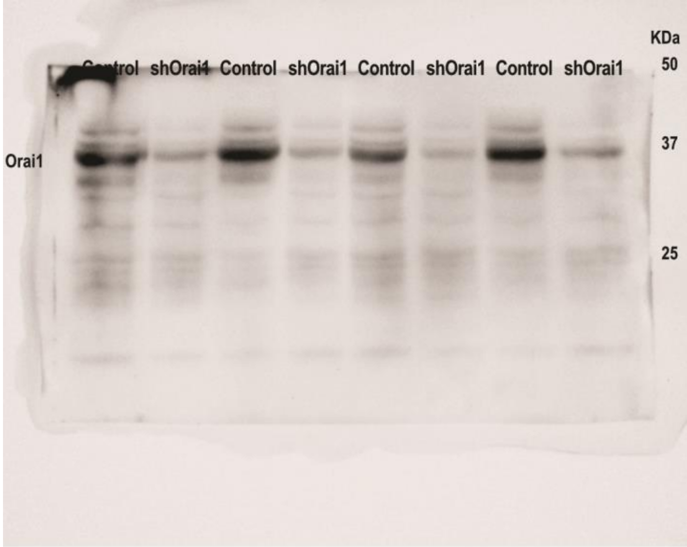

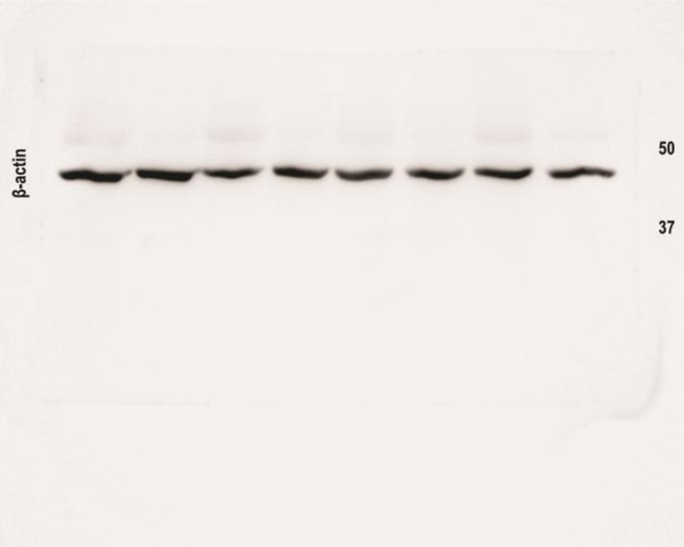

	Control	shOrai1	Control	shOrai1	Control	shOrai1	Control	shOrai1
Orai1	41603.4	9051.1	32479.7	6121.7	20412.2	7432.6	32686.4	10004.0
Beta actin	34603.8	37395.4	29410.4	28465.1	27211.2	27564.7	31377.1	25631.0
Orai/Bactin	**1.202**	**0.242**	**1.104**	**0.215**	**0.750**	**0.270**	**1.042**	**0.390**

	Mean	s.e.m
**Control**	1.025	0.097
**shOrai1**	0.279	0.039

Four samples, corresponding to 4 independent cultures non treated (Control) or transfected (shOrai1) with silencing plasmid shOrai1 were used to perform WB. The Table shows individual readings (OD) for each band and the normalized signal, as well as the average (and s.e.m) value for Control and transfected samples.
