# Supplementary figures and images for: Secretion of Interleukin 6 in Human Skeletal Muscle Cultures Depends on Ca2+ Signalling
Source: Biology (Basel). 2023 Jul 7;12(7):968. doi: 10.3390/biology12070968 (PMC10376320; doi:10.3390/biology12070968)

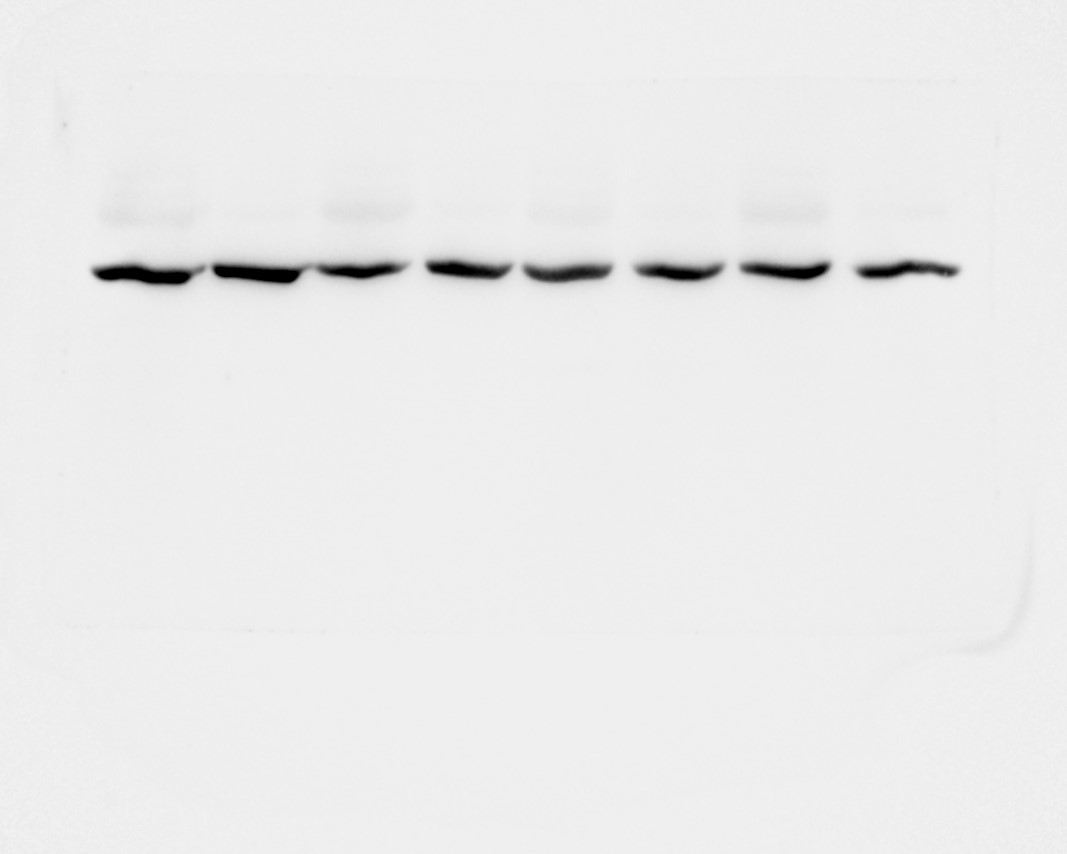

Supplement: Supplementary file 1 [file biology-12-00968-s001.zip › biology-2347404-original blott figures/Raw W Blott Beta actin ID2347404.tif]

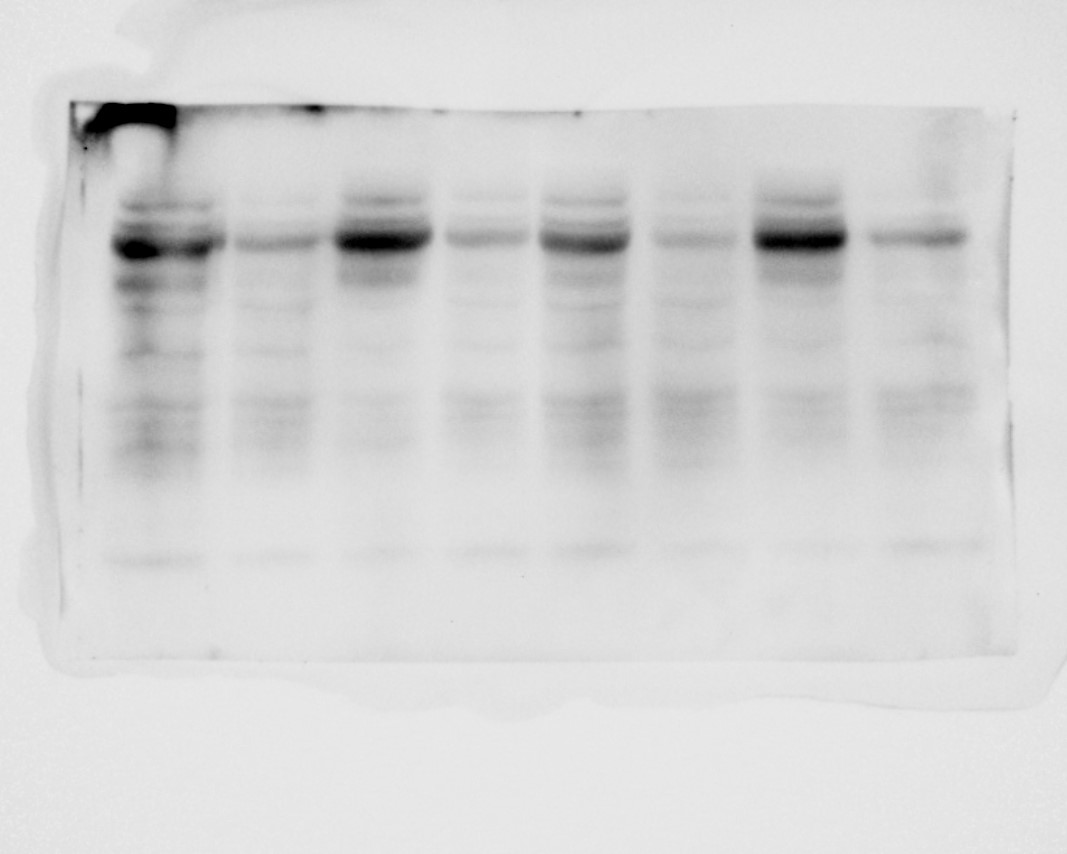

Supplement: Supplementary file 1 [file biology-12-00968-s001.zip › biology-2347404-original blott figures/Raw W Blott shOrai1 ID2347404.tif]

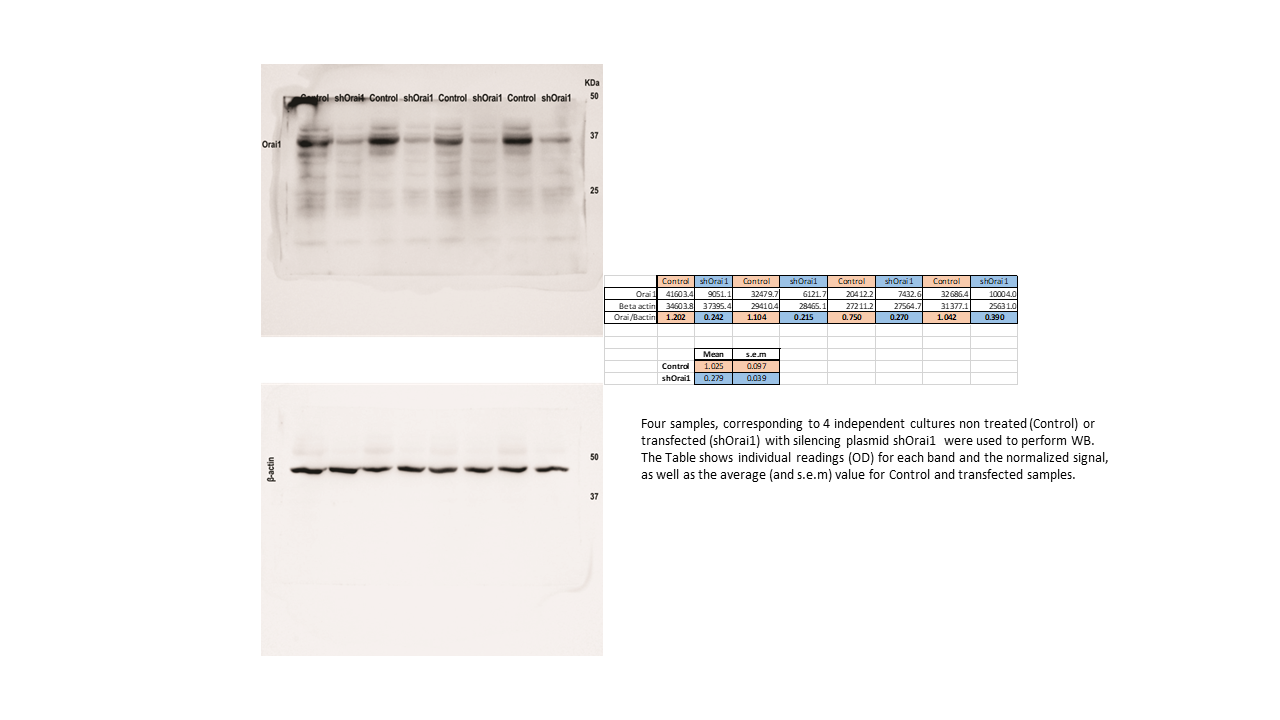

Supplement: Supplementary file 1 [file biology-12-00968-s001.zip › biology-2347404-original blott figures/Suppl Fig 2.tif]
